# Construction and characterization of an infectious cDNA clone of turtle grass virus X from a naturally infected *Thalassia testudinum* plant

**DOI:** 10.1128/mbio.02828-24

**Published:** 2024-12-11

**Authors:** Luis Alvarado-Marchena, Bradley T. Furman, Mya Breitbart

**Affiliations:** 1 College of Marine Science, University of South Florida, St. Petersburg, Florida, USA; 2 Florida Fish and Wildlife Conservation Commission, Florida Fish and Wildlife Research Institute, St. Petersburg, Florida, USA; The University of Tennessee Knoxville, Knoxville, Tennessee, USA

**Keywords:** turtle grass virus X (TGVX), infectious cDNA clone, agroinfection, marine plant virology, seagrass ecology, potexvirus

## Abstract

Seagrasses are a polyphyletic group of marine flowering plants that play crucial roles in nearshore ecology, yet their interactions with viruses remain largely unexplored. This study presents the construction and characterization of an infectious cDNA clone of the potexvirus turtle grass virus X (TGVX). The complete genome of this positive-sense single-stranded RNA virus was amplified from field samples of *Thalassia testudinum* and assembled into a pLX-based mini binary vector using a multi-fragment directional cloning strategy, resulting in the infectious clone pLX-TGVX. Agroinfection assays of potexvirus-free *T. testudinum* plants resulted in systemic infections by TGVX, as confirmed by multiplex RT-PCR experiments and phenotypic changes reflecting virus-induced symptoms. Ultrastructural studies also demonstrated significant cytopathological changes resulting from TGVX infection, including chloroplast swelling, reduced thylakoid grana, and the presence of viral replication organelles and filamentous virus-like particles. The development of the TGVX infectious clone offers a novel tool for investigating the impact of this virus on seagrass health and productivity. This study demonstrates the first successful agroinfection of a marine plant with an infectious clone, creating a new avenue for studying viruses identified through sequence-based surveys and paving the way for exploring the ecological significance of viral infection in these critical marine ecosystems.

**IMPORTANCE:**

This study pioneers the construction of an infectious clone of turtle grass virus X and describes its application in the natural marine plant host, *Thalassia testudinum*. The creation of this infectious clone not only provides a valuable tool for marine plant virology research but also opens new avenues for exploring the influence of viral infections on the health and productivity of seagrass meadows. Given that seagrasses play a crucial role in sediment stabilization, nutrient cycling, and habitat provisioning, understanding the impact of viruses on these ecosystems is essential for their effective conservation and management. This methodological advance enables detailed studies of viral replication, virus-host interactions, and the broader ecological implications of viral infections in marine plants.

## INTRODUCTION

Seagrasses, the only marine angiosperms, play crucial roles in marine ecology (1). Distributed along coastlines worldwide, these aquatic plants serve as feeding grounds, refuges, and nurseries for a wide variety of marine species (2, 3). They significantly contribute to sediment stabilization and nutrient cycling, helping to mitigate the effects of eutrophication and climate change (4–7). Additionally, seagrass ecosystems are vital for human economies as they support commercial and recreational fisheries and protect coastlines from erosion (8, 9). However, seagrass meadows have been in decline over the past few decades due to various anthropogenic factors such as pollution, eutrophication, coastal development, and climate change (2, 4, 10, 11).

Previous studies have determined that the seagrass microbiome is a key determinant of plant health and productivity (12–15). Microbial communities play significant roles in nutrition, disease resistance, and adaptation to environmental changes (16, 17). However, most research has focused on archaea, bacteria, and fungi, leaving the role of viruses largely unexplored (18).

*Thalassia testudinum* Banks ex König, also known as turtle grass, is a climax seagrass species in the Caribbean and the Gulf of Mexico (19). This plant not only provides habitat and food for various marine organisms but also plays a crucial role in stabilizing marine sediments (20). We previously identified a novel positive-sense single-stranded RNA virus, turtle grass virus X (TGVX, *Potexvirus*), in apparently healthy *T. testudinum* plants (18). Widespread viral infection in healthy plants has been demonstrated in terrestrial systems (21); however, this finding in seagrass meadows raises questions about viral dynamics and potential ecological effects.

Understanding and characterizing viral infections in seagrasses is crucial, as it can reveal how viruses influence plant health, resilience, and productivity (18). Infectious clones are a powerful tool that has revolutionized plant virology, allowing detailed studies on viral replication, gene expression, and virus-host interactions under controlled conditions (22, 23). In the context of marine plants, the use of infectious clones could also provide valuable information on how viruses affect host physiology and ecology.

Given the importance of *T. testudinum* in the dynamics of marine ecosystems and the scarcity of knowledge regarding viral infection, this study aimed to develop an infectious clone of TGVX capable of infecting turtle grass under controlled conditions to enable experimental research on this virus. Agroinfection (23) of marine plants with infectious clones will be transformative for understanding the role of viral infections in seagrass ecology and enable future comparisons of viral replication, evolution, and ecological impacts in terrestrial versus marine angiosperms.

## RESULTS

### Assembly of the TGVX infectious clone

**Fig 1:**
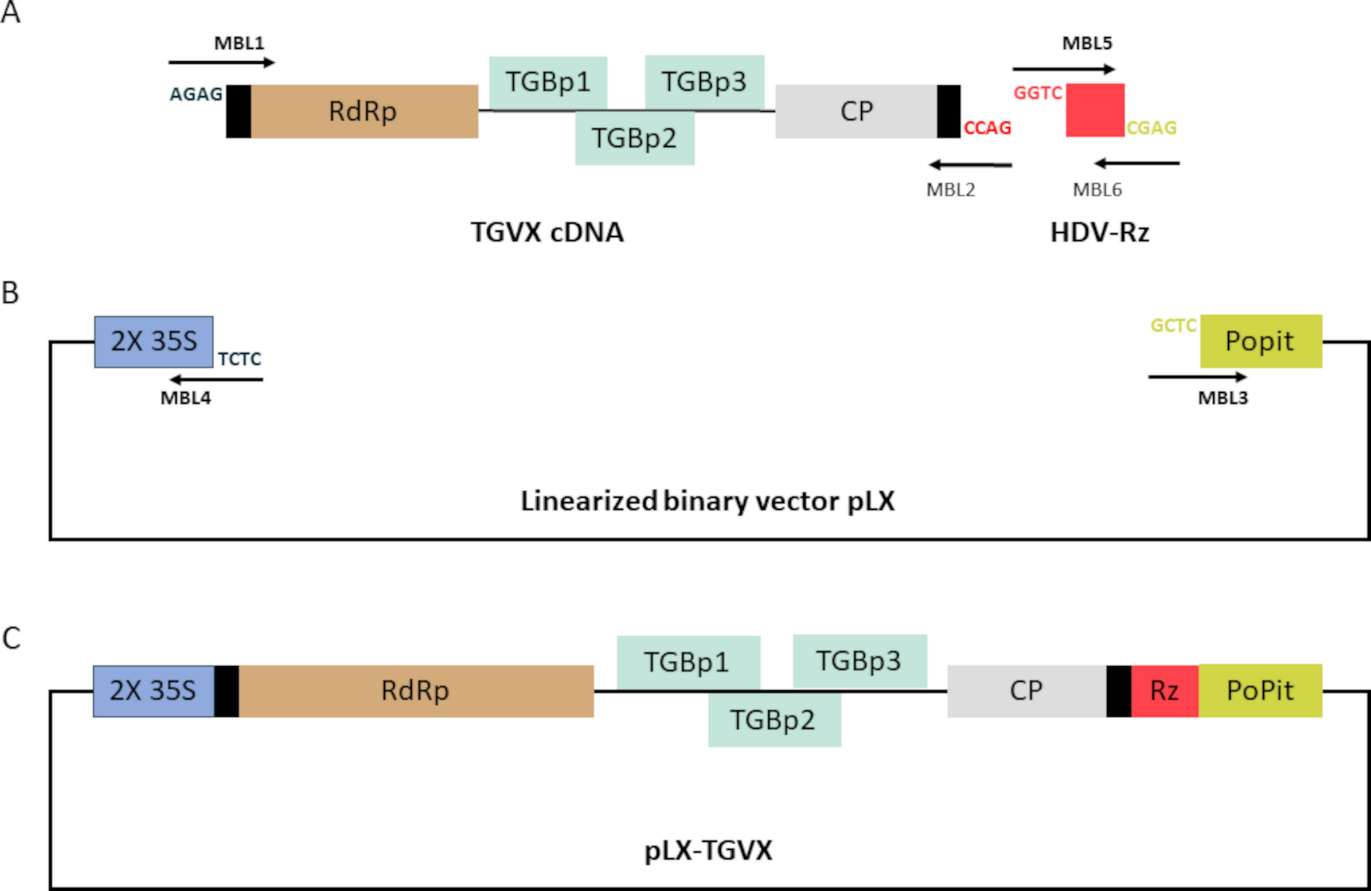
Assembly of a full-length TGVX infectious cDNA clone using a directional cloning strategy with the BsmBI restriction endonuclease. (**A**) TGVX cDNA and HDV-Rz possess specific cohesive overhangs, facilitating their fusion, (**B**) as well as their integration with the linearized binary vector. (**C**) Schematic diagram of the pLX-TGVX. Black boxes represent TGVX-UTR regions.

Since TGVX was sequenced directly from seagrass collected in Tampa Bay using viromics and no isolate is available, the creation of an infectious clone is crucial for advancing research on this virus. Assembly of the TGVX infectious clone (pLX-TGVX) was achieved using a multi-fragment directional cloning strategy with the BsmBI [CGTCTC(N^1^/N_5_)^^^] restriction endonuclease. This method allowed for precise integration of the TGVX cDNA (6.3 kb) and the Hepatitis delta virus ribozyme (HDV-Rz; 96 bp) into the linearized pLX binary vector (4.3 kb) through their specific cohesive ends (Fig. 1).

Complete plasmid sequencing revealed that the assembled TGVX genome in the pLX-TGVX clone (GenBank accession number PP887953) exhibited 96.1% identity with the reference TGVX genome (MH077559; Van Bogaert et al. [18]). The two genomes were colinear, with no significant indels detected.

### Infectivity of the TGVX infectious clone in *T. testudinum*

**Fig 2:**
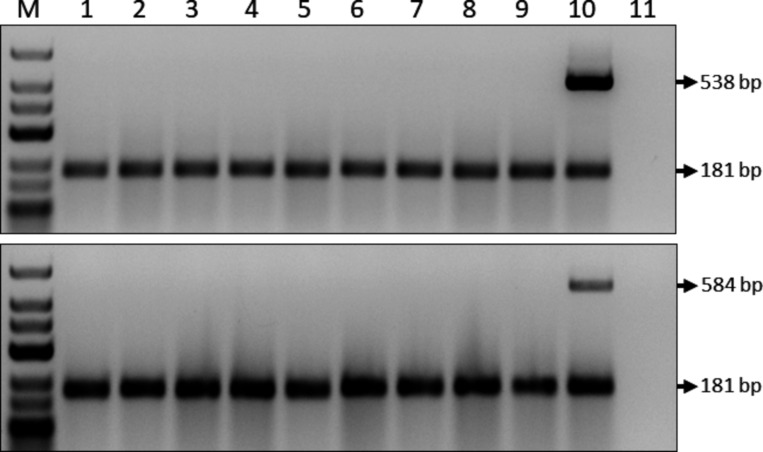
Representative results of multiplex RT-PCR confirming the absence of TGVX and other potexviruses in *T. testudinum* plants used for agroinfection assays. The upper gel shows the RT-PCR assay for TGVX CP (538 bp), and the lower gel shows the general RT-PCR assay for potexviruses (584 bp). In both gels, the band at 181 bp corresponds to the amplification of the mitochondrial *nad5* gene mRNA, which was used as an internal control. Size marker (Lane M): GeneRuler Low Range DNA Ladder (Invitrogen). Lanes 1–9: RT-PCR products from total RNA of *T. testudinum* plants. Lane 10: Positive control. Lane 11: Negative control (H_2_O).

To demonstrate infectivity of the pLX-TGVX clone, infectivity assays were performed in triplicate, with each independent replicate consisting of three field-collected *T. testudinum* plants inoculated with either the pLX-TGVX construct or the empty vector (MOCK). Prior to agroinfection, multiplex RT-PCRs were conducted using the TGVX coat protein (CP) and degenerate potexvirus replicase primers to confirm the absence of TGVX and any other potexviruses in the experimental plants transferred to the aquaria (Fig. 2). Once the absence of potexviruses was confirmed, agroinfiltration was performed.

**Fig 3:**
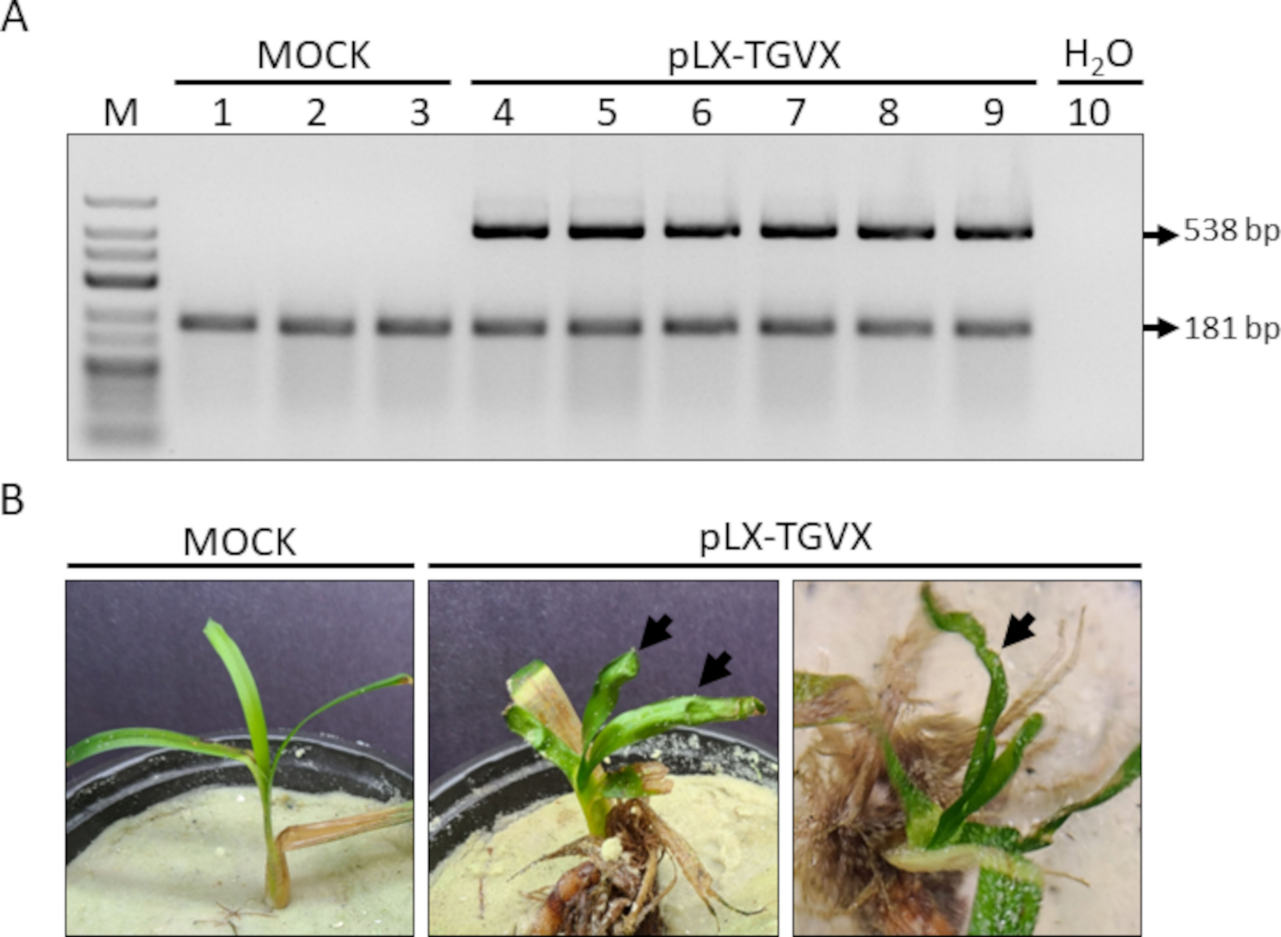
Representative results of molecular and phenotypic analyses of *T. testudinum* plants at 17 dpa. (**A**) Multiplex RT-PCR results using TGVX CP primers (538 bp) and the mitochondrial *nad5* gene mRNA (181 bp). Size marker (Lane M): GeneRuler Low Range DNA Ladder (Invitrogen). Lanes 1–3: RT-PCR products from mock-inoculated plants. Lanes 4–9: RT-PCR products from pLX-TGVX inoculated plants. Lane 10: Negative control. (**B**) Phenotypes shown by a mock-inoculated plant (left) and two different TGVX-positive plants (middle and right) with deformed, wrinkled, or wavy-edged leaves (black arrows).

Multiplex RT-PCR results using the TGVX CP primers, performed at 17 days post agroinfiltration (dpa), confirmed the presence of TGVX in the upper, non-inoculated leaves of plants agroinfected with pLX-TGVX (Fig. 3A). TGVX-positive plants showed distinct symptoms of viral infection, including deformed, wrinkled, or wavy-edged leaves (Fig. 3B). In contrast, no TGVX viral RNA (vRNA) or symptoms of viral infection were detected in the mock-inoculated plants (Fig. 3A and B). These results were consistently observed across three independent trials, involving a total of 18 plants.

### Ultrastructural analyses of TGVX infection

**Fig 4:**
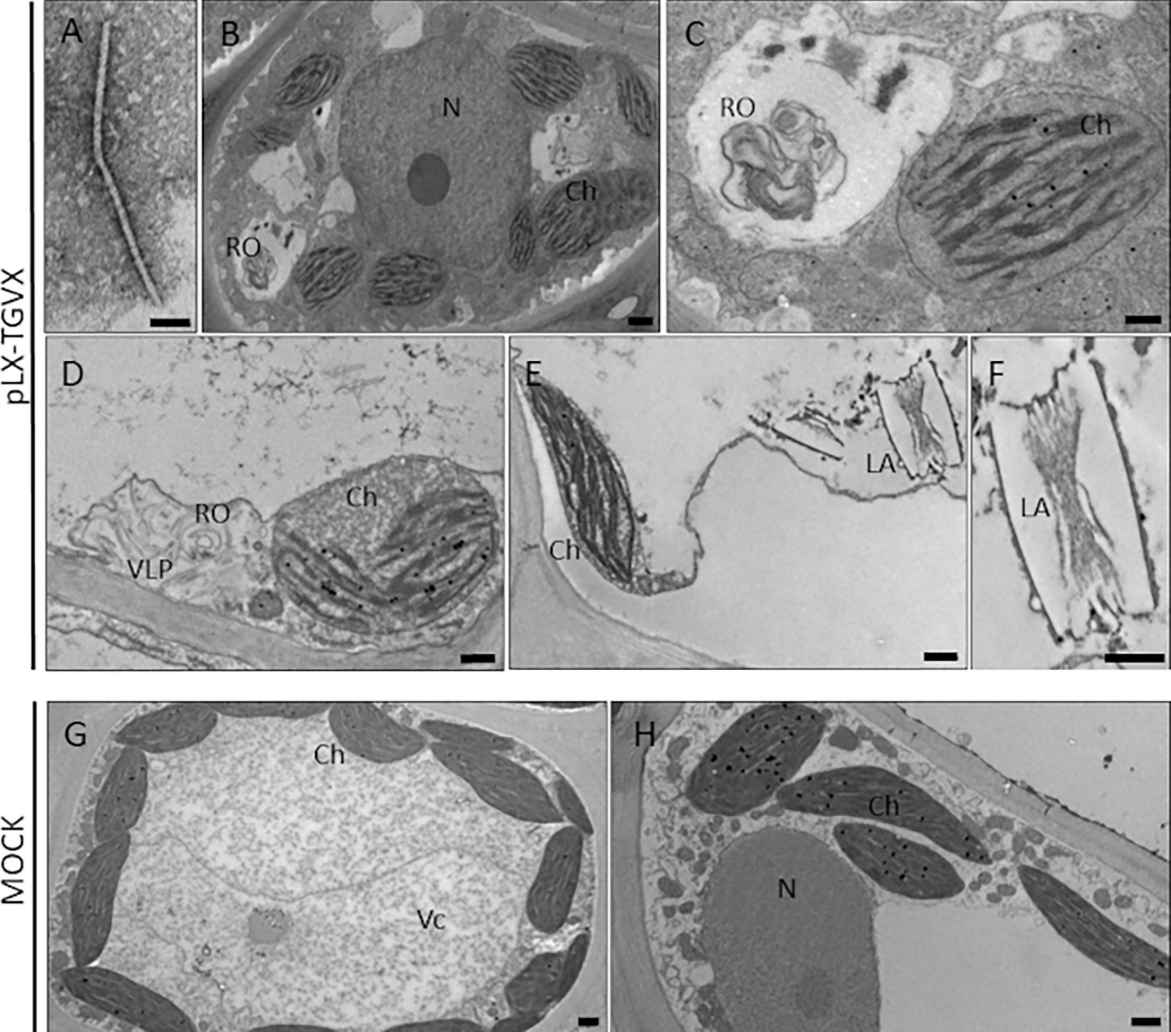
Ultrastructural analyses of *T. testudinum* plants infected by TGVX following infiltration with the pLX-TGVX construct, as well as the control plants that were mock inoculated. (**A**) Filamentous VLP of TGVX, visualized using the leaf dip method. Scale bar: 50 nm. Panels (**B–D**) show cytopathological changes in chloroplasts (Ch) and the presence of viral replication organelles (ROs). In panel (**D**), TGVX VLPs are closely associated with these ROs. Panels (**E and F**) illustrate the formation of laminar aggregates (LA), a hallmark of potexvirus infection. Panels (**G and H**) depict the ultrastructure of mock-inoculated plants, which maintain normal chloroplast morphology with dense thylakoid grana and show no signs of viral cytopathology. The nuclei (N) and vacuole (Vc) are also indicated. Scale bars: 1 µm for panels B–H.

Van Bogaert et al. (18) previously documented virus-like particles (VLPs) from TVGX-positive seagrasses, which were consistent in morphology with known potexviruses. However, those transmission electron micrographs originated from crude virus preparations of seagrass leaves, and the imaged particles were not directly linked to TGVX. Here, the leaf dip method visualized filamentous virions typical of potexviruses only in plants agroinfiltrated with the infectious TGVX clone. These VLPs displayed a consistent diameter of 13 nm and an average length of 470 nm (Fig. 4A) (24).

Agroinfection of *T. testudinum* with the TGVX infectious clone also allowed for the first examination of the impacts of viral infection on host physiology. Ultrastructural analyses of *T. testudinum* plants agroinfected with pLX-TGVX revealed significant cytopathological changes in non-inoculated leaves. In all analyzed samples, an alteration in chloroplast cyclosis was observed (Fig. 4B). Additionally, the chloroplasts appeared swollen and exhibited a significant reduction in thylakoid grana (Fig. 4B through E). Viral replication organelles (RO) with convoluted membranes were also identified (Fig. 4B through D) (25), along with putative VLPs associated with these ROs (Fig. 4D). Laminar aggregates were also observed in some infected tissues (Fig. 4E and F), consistent with previous observations of potexvirus infections (26, 27). Notably, mock-inoculated plants did not exhibit any symptoms of infection or similar cytopathological changes (Fig. 4G and H).

In summary, our results demonstrate that the infectious TGVX clone can induce systemic infection in *T. testudinum* plants, as evidenced by the detection of vRNA and characteristic cytopathological changes in non-inoculated leaves.

## DISCUSSION

Our results demonstrate the successful construction, application, and characterization of an infectious clone of TGVX (pLX-TGVX, Fig. 1) capable of systemically infecting *T. testudinum* plants. The infectivity of pLX-TGVX was confirmed through various methods. Multiplex RT-PCR validated the presence of the virus in non-inoculated tissues of agroinfected plants (Fig. 3A), while phenotypic analysis revealed the presence of distinctive symptoms of viral infection (Fig. 3B). Transmission electron microscope (TEM)-based observations showed filamentous viral particles typical of potexviruses in leaf extracts of the agroinfiltrated plants (Fig. 4A). Significant cytopathological changes in chloroplasts and the formation of viral replication organelles were also observed in non-inoculated leaves of agroinfected plants (Fig. 4B through D). These findings are consistent with previous studies on the cytopathology of potexvirus infections in terrestrial plants (26, 27).

The number of reported viruses in seagrasses is increasing, emphasizing the need for molecular tools that enable detailed studies of viral replication and virus-host interactions in these marine angiosperms. Recent studies have significantly expanded our knowledge of seagrass virology, identifying 14 viruses across five different seagrass species. In addition to TGVX in *T. testudinum* (18), *Cymodocea nodosa* hosts three viruses from the family *Betaflexiviridae* (28, 29), while six viruses have been discovered in *Zostera marina*, including Zostera associated varicosavirus 1 from the family *Virgaviridae* (30), three alphaendornaviruses from the family *Endornaviridae* (29–31), and two members of the family *Amalgaviridae* (29). *Zostera muelleri* harbors three additional viruses from the families *Endornaviridae*, *Virgaviridae*, and *Betaflexiviridae*, respectively (29, 32). Finally, in *Zostera japonica*, a novel strain of Cucumber mosaic virus (CMV-Zja) from the family *Bromoviridae* has been identified (29). These findings underscore the diversity and ecological significance of the seagrass virome. However, while these prior studies have identified the presence of viruses in seagrasses, our work is the first to create an infectious clone capable of systemic infection of seagrass through agroinfection. This advanced methodology provides new opportunities to investigate how viruses affect the physiology and productivity of seagrasses, while enabling detailed studies of viral replication, evolution, and ecological impacts (33).

The ecological implications of TGVX infection in *T. testudinum* remain unknown. Our findings demonstrate that TGVX infection can induce significant cytopathological changes in plant leaves, potentially impacting the health and productivity of seagrass meadows. Although the infection of experimental plants described here resulted in deformed and wavy-edged leaves, these symptoms have not been observed in naturally infected plants at our research field site. This disparity is not surprising since various abiotic (temperature, salinity, and nutrient levels) and biotic (plant stressors, other members of the microbiome) factors differ between the seagrass experimentally agroinfected in the laboratory under optimal conditions and those found naturally in the field. Given that marine flowering plants, including *T. testudinum*, provide valuable ecosystem services dependent in part on high productivity (1, 3, 4, 34, 35), understanding the effects of viral infection on their health is essential. If TGVX is proven pathogenic under natural conditions, it may pose a potential risk to seagrass ecosystem function or resilience. However, it should be noted that many plant viruses do not negatively impact their hosts, and in fact, some can be beneficial by providing resistance to other pathogens and environmental stressors (36). Future studies using the technique developed here can explore the transcriptional response of *T. testudinum* to TGVX infection and assess changes in TGVX-host dynamics under different environmental conditions (e.g., varied temperatures and salinities). Additionally, the infectious clone can be used to determine how TGVX interacts with the *T. testudinum* microbiome, an ecologically important topic given the significant role of microbial communities in nutrition, disease resistance, and adaptation to environmental changes.

In conclusion, our study provides a novel tool for studying viral infections in seagrasses, a transformative advance that will allow seagrass virology to expand from sequence-based discovery to studying physiological responses and ecological effects. The ability to manipulate and study viral infections under controlled conditions will enable advances in understanding the complex roles that viruses play in marine plant ecology. This knowledge could inform conservation strategies and assist in the management of seagrass meadows, ensuring their health and resilience in the face of environmental changes.

## MATERIALS AND METHODS

### Viral source

Naturally infected *T. testudinum* plants were collected at the Terra Ceia Aquatic Preserve in Tampa Bay, Florida, USA, at sampling point N01 (GPS coordinates: 27°35'01.5"N 82°36'58.1"W). This location was previously identified by Van Bogaert et al. (18) as having a high prevalence of TGVX.

### Virus isolation, RNA extraction, and cDNA synthesis

Viral particles were isolated from TGVX-positive leaf tissue following the protocol described by Sánchez-Navarro et al. (37). Briefly, the tissue was macerated in a mortar at room temperature with two volumes of freshly prepared extraction buffer (100 mM K_2_HPO_4_, 100 mM ascorbic acid, and 20 mM EDTA, adjusted to pH 7.1). The macerate was filtered through Miracloth, and 1 mL aliquots were transferred to microcentrifuge tubes. Each aliquot was mixed with 0.5 volumes of chloroform:butanol (1:1) and vortexed for 30 seconds. Following centrifugation at 6,300× *g* (RCF) for 15 minutes at 4°C, the supernatant was transferred to a new tube and mixed with 0.2 volumes of 30% PEG-20,000. After incubating at 4°C for 20 minutes, the mixture was centrifuged again at 6,300× *g* (RCF) for 20 minutes at 4°C. The resulting pellet was resuspended in 250 µL of 1× phosphate buffer (10 mM NaH_2_PO_4_, 1 mM EDTA, pH 7.0) and stored at −80°C.

RNA extraction was performed using the Quick-RNA Plant Miniprep Kit (Zymo Research) according to the manufacturer’s instructions. To amplify the full genome of TGVX, RT-PCR was conducted using the SuperScript IV One-Step RT-PCR System (Invitrogen). The reaction mixture contained 25 ng of vRNA, 25 µL of 2× Platinum SuperFi RT-PCR Master Mix, 0.5 µL of SuperScript IV RT Mix, and 0.2 µM of MBL1 and MBL2 primers (Table S1). These primers were designed based on the complete TGVX genome (GenBank accession number MH077559) previously reported by Van Bogaert et al. (18). Cycling parameters were as follows: reverse transcription at 50°C for 10 minutes, RT inactivation/initial denaturation at 98°C for 2 minutes, followed by 30 cycles of denaturation at 98°C for 10 seconds and annealing/extension at 72°C for 3.15 minutes. The final extension step was carried out at 72°C for 5 minutes. The amplified products were extracted from agarose gels and purified using the GeneJET PCR Purification Kit (ThermoFisher).

### Cloning the TGVX cDNA

To assemble the TGVX infectious clone, a multi-fragment directional cloning strategy was employed using the class IIS restriction endonuclease BsmBI (38). A pLX-based mini binary vector was used as the backbone (39, 40). This vector includes the 1.5 kb pBBR1 origin, two copies of the Cauliflower mosaic virus 35S promoter and the Potato Protease inhibitor II terminator (PoPit; Fig. S1). First, the vector was linearized by inverse PCR using the MBL3 and MBL4 primers (Table S1; Fig. S1). Platinum SuperFi II DNA Polymerase was used for this step, following the manufacturer’s instructions. The linearized pLX binary vector was also treated with DpnI (ER1701, ThermoFisher) to remove plasmid templates. Additionally, an optimized variant of the HDV-Rz was synthesized using the overlap extension PCR technique. This involved using the MBL5 and MBL6 primers (Table S1), which overlap at their 3′ ends to allow for seamless extension and synthesis of the ribozyme, following the method described by Schürer et al. (41). The resultant amplified fragments were extracted from the agarose gel using the GeneJET PCR Purification Kit (ThermoFisher). The (RT-) PCR products (TGVX cDNA, HDV-Rz and the backbone) were then subjected to digestion with BsmBI (ER0452, ThermoFisher) followed by a single ligation reaction using T4 DNA Ligase (Promega) according to the manufacturer’s instructions. Ligation products were transferred in *Escherichia coli* strain DH5α (C449601, Thermofisher) by heat-shock at 42°C. Positive clones were screened by colony PCR. Whole plasmid sequencing was conducted using Oxford Nanopore technology (Eurofins Genomics LLC, USA).

### Plant collection, transplantation, and aquarium growth conditions

Young *T. testudinum* plants were collected from Lassing Park in St. Petersburg, Florida, USA (GPS coordinates: 27°45'13.7"N 82°37'41.1"W) for agroinfection assays. This particular site was chosen because of the extremely low incidence of TGVX; therefore, we expected that the collected plants would not contain TGVX. The plants were individually transported in seawater to the Knight Oceanographic Research Center Aquarium at the University of South Florida, St. Petersburg Campus. Each plant was then transplanted into a 6-cm diameter pot filled with beach sand and placed within a 10 L tank filled with artificial seawater. Instant Ocean (42) was used to maintain a salinity of 30 psu (±0.5). Each tank was supplied with continuous aeration, and temperature was maintained at 30°C (±0.5°C) using a water bath. The plants were subjected to a photoperiod of 16 hours light and 8 hours dark. Water level and salinity within each tank were monitored every 24 hours. Prior to agroinfection, the plants were acclimated to the experimental conditions for 2 days.

### Detection of vRNAs

Prior to agroinfection, RT-PCR analyses were conducted to confirm the absence of TGVX and any other potexviruses in the experimental plants. Two different multiplex RT-PCR assays were employed using the SuperScript IV One-Step RT-PCR System (Invitrogen). Specific primers (MBL5/MBL6) targeting the CP were designed for TGVX detection (Table S1), while degenerate replicase primers (Potex-5/Potex-2RC) were used for detecting other potexviruses (43, 44). Additionally, primers for the mRNA mitochondrial *nad5* gene (45) were included as an internal control. The reaction mixture consisted of 150 ng of total RNA, 5 µL of 2× Platinum SuperFi RT-PCR Master Mix, 0.1 µL of SuperScript IV RT Mix, and either 0.2 µM of CP primers or 0.35 µM of potexvirus degenerate replicase primers, plus 0.2 µM of *nad5* gene primers. The thermal cycling conditions were as follows: reverse transcription at 50°C for 10 minutes, inactivation/initial denaturation at 98°C for 2 minutes, followed by 30 cycles of denaturation at 98°C for 10 seconds, annealing at 60°C for 30 seconds and extension at 72°C for 18 seconds. A final extension step was conducted at 72°C for 5 minutes. The amplified products were analyzed by electrophoresis on 2.0% agarose gels pre-stained with ethidium bromide in 1× TAE buffer for 35 minutes at 100 V. A GeneRuler Low Range DNA Ladder (Invitrogen) with markers at 25, 50, 75, 100, 150, 200, 300, 400, 500, and 700 bp was run alongside samples. Specific bands corresponding to the TGVX CP (538 bp), degenerate potexvirus primers (584 bp), and the mRNA of the mitochondrial *nad5* gene (181 bp) were visualized under ultraviolet light. Each RT-PCR run included positive and negative (water) controls to ensure the accuracy of the results.

### Plant infection

For agroinfection experiments, *Agrobacterium tumefaciens* C58C1 strains (1086–18, Genesee Scientific Co.) carrying either the pLX-TGVX construct or the empty vector (employed as a mock treatment) were grown in Luria-Bertani broth containing rifampicin (50 µg/mL) and kanamycin (100 µg/mL) for 24 hours at 28°C with agitation at 128 rpm. Bacterial cells were harvested by centrifugation and resuspended in induction buffer (10 mM MES pH 5.6, 10 mM MgCl_2_, and 200 µM acetosyringone) to a final OD_600_ of 0.6. The bacterial suspension was then incubated at room temperature with gentle agitation for 3 hours. *T. testudinum* plants were agroinfiltrated on the abaxial side of two leaves using a 1 mL needle-less syringe. Detection of vRNAs by RT-PCR and TGVX visualization using electron microscopy was performed 17 dpa on the upper non-inoculated leaves.

### TGVX visualization

TGVX-like particles were visualized using the leaf dip method developed by Brandes and Wetter (46). A drop of infected sap, obtained by squeezing the freshly cut surface of a leaf, was applied to a nickel grid (Ted Pella Inc., catalog number 01800 N-F) and incubated at room temperature for 10 minutes. Excess sap was then removed using Whatman filter paper. The grids were stained with 10 µL of 10% gadolinium acetate tetrahydrate (Ted Pella Inc., catalog number 19485) for 10 minutes and then dried in a chemical fume hood for 1 hour.

Viral cytopathology was examined following the protocol established by Alvarado et al. (47). Briefly, the tissue from TGVX-infected and mock-inoculated plants was fixed for 2 days at 4°C in 2.5% glutaraldehyde and 2% paraformaldehyde dissolved in 0.1 M phosphate buffer (pH 7.2), followed by post-fixation in 2% osmium tetroxide. The samples were dehydrated in gradual acetone dilutions and embedded in low-viscosity Spurr resin. Plant tissues and TGVX-like particles were examined with a JEOL JEM 1400 TEM operating at 80 kV.

## SUPPLEMENTAL MATERIAL

Supplemental material mbio.02828-24-s0001.docx Table S1; Fig. S1.

ASM does not own the copyrights to Supplemental Material that may be linked to, or accessed through, an article. The authors have granted ASM a non-exclusive, world-wide license to publish the Supplemental Material files. Please contact the corresponding author directly for reuse.
