# Supplementary material for: Construction and characterization of an infectious cDNA clone of turtle grass virus X from a naturally infected Thalassia testudinum plant
Source: mBio. 2024 Dec 11;16(1):e02828-24. doi: 10.1128/mbio.02828-24 (PMC11708015; doi:10.1128/mbio.02828-24)
Supplement: Supplemental material — Table S1; Fig. S1. [file mbio.02828-24-s0001.docx]

**SUPPLEMENTARY MATERIAL**

**Table S1** Sequences of the oligonucleotides used in the (RT-) PCR in this work. The underlined nucleotides in the primers MBL1-6 indicate the recognition site for the BsmBI [CGTCTC(N^1^/N_5_)^] restriction endonuclease. The bold nucleotides in the primers MBL5 and MBL6 correspond to the overlapping regions that allow for the seamless extension and synthesis of the HDV-Rz.

| **Primers** | **Sequence (5’-3’)** | **Purpose (Product size)** |
| --- | --- | --- |
| MBL1_s | CGTCTCGAGAGGGAAAACTCTTCCACAACTGAACCC | RT-PCR TGVX cDNA  (6.3 kb) |
| MBL2_as | CGTCTCCGACCCTTTTTTTTTTTTTTTTTTTTTTTTTTAAAGTGATGTGTGCATTC |  |
| MBL3_s | CGTCTCCGCTCCCTAGACTTGTCCATC | pLX inverse PCR  (4.3 kb) |
| MBL4_as | CGTCTCCCTCTCCAAATGAAATGAACTTCC |  |
| MBL5_s | CGTCTCGGGTCGGCATGGCATCTCCACC**TCCTCGCGGTCCGACCTGGGCTA** | HDV-Rz overlap extension PCR  (93 bp) |
| MBL6_as | CGTCTCGGAGCGCTTCTCCCTTAGCCTACCGAAG**TAGCCCAGGTCGGACCGCGAGGA** |  |
| MBL5_s | CACAGATGAAGAGCTGACC | TGVX CP primers for RT-PCR multiplex  (181 bp) |
| MBL6_as | TTCGATGAAGTAAGTGGCGG |  |
| Potex-5 | CAYCARCARGCMAARGAYGA | Degenerate potexvirus primers for RT-PCR multiplex  (584 bp)  **Van der Vlugt & Berendsen** (43) |
| Potex-2RC | AGCATRGCNSCRTCYTG |  |
| nad5_s | GATGCTTCTTGGGGCTTCTTGTT | Mitochondrial *nad5* primers for RT-PCR multiplex  (181 bp)  **Menzel et al.** (45) |
| nad5_as | CTCCAGTCACCAACATTGGCATAA |  |

**
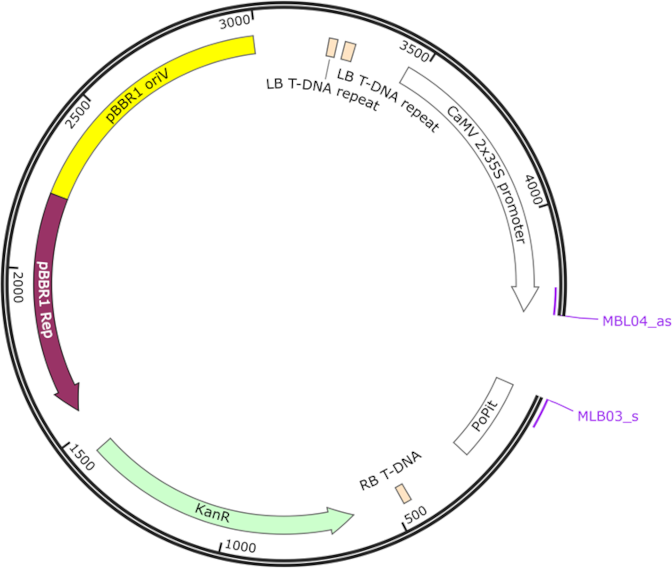
**

**FIG S1** Schematic diagram of the pLX-based mini binary vector used in this study. Length: 4.4 kb
